# Association of priori-defined DASH dietary pattern with metabolic health status among Iranian adolescents with overweight and obesity

**DOI:** 10.1038/s41598-024-55749-4

**Published:** 2024-02-29

**Authors:** Hajar Heidari, Saeideh Mirzaei, Ali Asadi, Masoumeh Akhlaghi, Parvane Saneei

**Affiliations:** 1 Nutrition and Food Security Research Center, Isfahan University of Medical Sciences, Isfahan, Iran; 2 Department of Community Nutrition, School of Nutrition and Food Science, Shiraz University of Medical Sciences, Shiraz, Iran; 3 Department of Exercise Physiology, School of Physical Education and Sport Sciences, University of Tehran, Tehran, Iran; 4 Department of Community Nutrition, School of Nutrition and Food Sciences, Shiraz University of Medical Sciences, Shiraz, Iran; 5 Department of Community Nutrition, School of Nutrition and Food Science, Nutrition and Food Security Research Center, Isfahan University of Medical Sciences, PO Box 81745-151, Isfahan, Iran

**Keywords:** Obesity, Metabolic health status, DASH diet, Adolescents, Metabolic disorders, Nutrition

## Abstract

There was no evidence on the relationship of Dietary Approaches to Stop Hypertension (DASH) with metabolic health condition in adolescents with overweight and obesity. The purpose of this research was to investigate the association of priori-defined DASH dietary pattern with metabolic health status among adolescents with overweight and obesity in Iran. A cross-sectional survey performed on a representative sample of adolescents with overweight and obesity (n = 203). Dietary intakes were collected via a validated food frequency questionnaire and DASH score was characterized according to eight components. Data of anthropometric measures, blood pressure, circulating insulin, fasting blood sugar, and lipid profile were collected. Metabolic health status was defined based on criteria of International Diabetes Federation (IDF) and insulin resistance (IR). Based on IDF and IDF/IR criteria, 38.9% and 33.0% of adolescents suffered from metabolically unhealthy overweight/obesity (MUO). After controlling all confounders, subjects in the highest vs. lowest tertile of DASH diet had respectively 92% and 91% lower odds of MUO based on IDF definition (OR = 0.08; 95%CI 0.03–0.22) and IDF/IR criteria (OR = 0.09; 95%CI 0.03–0.29). Subgroup analysis by sex and body mass index determined that this relationship was more powerful in girls and overweight individuals. Also, in fully adjusted model, highest vs. lowest adherence to DASH diet was linked to decreased odds of hyperglycemia (OR = 0.07; 95% CI 0.03–0.21), hypertriglyceridemia (OR = 0.26; 95% CI 0.09–0.73), low HDL cholesterolemia (OR = 0.30; 95% CI 0.12–0.73) and insulin resistance (OR = 0.07; 95% CI 0.02–0.28), as metabolic health components. Greater compliance to DASH dietary pattern was linked to a remarkable lower odd of metabolic unhealthy condition among Iranian adolescents, especially in overweight subjects and girls. More prospective surveys are required to assert these results.

## Introduction

Adolescent overweight and obesity have become a serious general health problem worldwide ^1^. It has been predicted that the number of children and adolescents with overweight and obesity will reach more than 400 million worldwide and 4 million in Iran by 2025 ^2,3^. Excess body mass index (BMI) in adolescents is related to metabolic disorders, including hypertension, blood lipid disorders, impaired glucose metabolism, and insulin resistance, all of which enhance the risk of developing cardiovascular diseases (CVD) and type 2 diabetes followed by premature mortality in the future. These conditions impose a significant burden on health care systems in addition to individuals and their families ^4,5^. The coexistence of obesity and metabolic disorders is defined as metabolically unhealthy overweight/obesity (MUO) ^6^, while some individuals have no metabolic abnormalities despite having excess fat in the body and are defined as metabolically healthy overweight/obese (MHO) ^7^.

Both genetics and lifestyle (including dietary habits and physical activity) are determinants of metabolic health status and can cause a shift from MHO to MUO or vice versa ^8^. Findings of recent investigations indicated positive associations between high consumption of vegetables and fruits and low sugary drinks and fats, as components of healthy dietary patterns with MHO ^6,9^. Among healthy dietary patterns, a diet emphasizing more consumptions of vegetables, fruits, whole grains, legumes, seeds, and low-fat dairy, as well as less consumption of processed and red meat, sodium and soft drinks are known as Dietary Approaches to Stop Hypertension (DASH) ^10^.

Numerous studies have investigated DASH diet in adolescence, but controversial findings were reported ^11–16^. In several epidemiological studies, no relationship was shown between DASH dietary pattern and overweight, obesity, and other cardio-metabolic variables in Brazilian ^12^, American ^13^ and Iranian ^15^ adolescents. Whereas in another investigation on adolescents, higher DASH score was linked to reduced general and central obesity risk, while no link was found in case of dyslipidemia risk ^14^. Results of two separate studies have found that DASH diet was negatively linked to BMI and abdominal obesity, hypertension, high fasting blood glucose, and metabolic syndrome (MetS) in children/adolescents ^11,16^. A clinical trial reported that compliance to DASH diet for 6 weeks could decrease the prevalence of hypertension in adolescents with MetS ^17^. Furthermore, a systematic review confirmed beneficial impacts of this pattern on hypertension, overweight and obesity in adolescents ^18^. Although, surveys in adults have shown a positive association between DASH diet and metabolic health ^19,20^, as far as we know, only Rahimi et al. have examined the association between DASH dietary pattern and MUO among Iranian overweight and obese children of elementary schools (6–13 years). Their findings indicated the beneficial effects of DASH diet on insulin sensitivity and metabolic health ^8^.

Considering the consequences of unhealthy metabolic obesity in the future life of adolescents, it seems that assessing the relationship between dietary patterns of adolescents with overweight and obesity and their metabolic health condition have notable importance. Since no study has been conducted on metabolic status in adolescents with overweight and obesity with DASH dietary pattern, the present investigation examined the association of priori-defined DASH diet with metabolic health status among adolescents with overweight and obesity in Iran.

## Materials and methods

### Participants and study design

We performed a cross-sectional survey in a representative sample of Iranian adolescents between the ages of 12 and 18 years. The sample size was determined based on previous articles ^21,22^, which have reported that almost 40–60% of the Iranian adolescents with overweight and obesity suffered from MUO. Hence, with a power = 80%, type I error = 0.05, desired confidence interval = 0.95%, and precision (d) = 7%, the number of samples was computed as 188 individuals. The participants included middle school and high school students were selected by a multi-stage cluster random sampling method. sixteen girls' and boys' schools of six different districts of Isfahan city were randomly chosen and body weight (kg) and height (cm) of all students of these schools were measured. Body mass index (BMI) was calculated using with the Quetelet formula (kg/m^2^); then, adolescents were classified as obese, overweight, and normal-weight ^23^. Using this approach, adolescents with overweight and obesity, from a wide range of social and economic statuses were included in this study. Students were considered eligible for inclusion if they were not on a weight-loss diet, had no diagnosed genetic or endocrine disease (for example hypothyroidism, type 1 diabetes mellitus, or Cushing syndrome), and were not using any mineral/vitamin supplements or any medicines that might influence their metabolic profile. Finally, we enrolled 203 adolescents with overweight or obesity, including 101 and 102 boys and girls, respectively. All subjects and their parents signed a written informed agreement prior to the investigation. The current survey protocol was authorized by the ethics committee of the Isfahan University of Medical Sciences with the ethical code: IR.MUI.PHANUT.REC.1402.068.

### Dietary intake assessment

Participants' dietary intakes during the past year were assessed in face-to-face interviews by two trained nutritionists, via a validated food frequency questionnaire (FFQ) containing 147 items ^24^. Former studies have shown that this FFQ has an acceptable validity and reliability to evaluate dietary intakes and their relationship with various diseases in Iranian adolescents ^25,26^. Subjects were asked to indicate whether they consumed each food item monthly, weekly or daily, and to indicate the amount consumed based on standard portion sizes. Afterward, household measurements were utilized to transform the portion sizes of consumed food items to g/day ^27^. Nutritionist IV software was used to calculate energy and nutrients daily intake. Although this software is based on the United States Department of Agriculture (USDA) food composition database, it has been reformed for some Iranian foods.

### Assessment of DASH dietary pattern

DASH score was created according to foods and nutrients emphasized or minimized in DASH dietary pattern, concentrating on eight components: higher consumption of whole-grains, low-fat dairy products, nuts, seeds and legumes, vegetables, and fruits and lower consumption of sweetened beverages, sodium, and red and processed meats ^28,29^. To compute a DASH score for each student, subjects were first categorized based on energy-adjusted quintile classifications of their dietary intake of the mentioned components. For whole-grains, vegetables, low-fat dairy products, fruits, nuts, seeds and legumes, those in the lowest quintile of intake obtained a score of 1, subjects in the 2nd, 3rd, 4th, and 5th quintiles got a score of 2, 3, 4 and 5, respectively. Regarding sweetened beverages, salt, and red and processed meats, subjects in the lowest quintile of intake have received the greatest score (a score of 5), while those with the highest quintile received a score of 1. At the end, the scores of eight components were summed up to obtain an adolescent's total DASH score ranged from 8 to 40. Students who received the greatest DASH score were more compliant with DASH dietary pattern.

### Assessment of anthropometric indicators and cardio-metabolic risk factors (CMRFs)

Height was recorded via a stadiometer to the closest 0.1 cm, while standing barefoot with shoulders in usual alignment. Weight was determined by a calibrated electronic scale to the closest 100 g while students were without any shoes and in light clothing. Then, BMI was computed as weight (kg)/ height^2^ (m^2^). Students were categorized as overweight (BMI between 85 and 95th percentile), and obese (BMI greater than 95th percentile) according to WHO growth curve of age-sex-specific BMI percentiles for adolescents ^23^. Waist circumference (WC) was gauged twice with a non-elastic tape to the closest 0.1 cm, and the average of these two was recorded as WC value. Systolic/diastolic blood pressure (SBP, DBP) were gauged twice, at least 15 min apart, on the right arm via a mercury sphygmomanometer with a proper cuff size. The mean of two gauges was presented as the individual’s SBP or DBP. To measure biochemical indices, venous blood samples were gathered after a minimum 12 h fasting. Fasting blood sugar (FBS), triglycerides (TG), and high-density lipoprotein cholesterol (HDL-c) were measured with standard methods. Insulin concentrations were measured by ELISA kits from Diagnostic Biochem Canada Inc. Homeostasis Model Assessment Insulin Resistance (HOMA-IR) was then calculated by imputing fasting insulin and FBS in the formula ^30^.

### Assessment of metabolic status

Two different strategies were applied to stratify adolescents into MUO and MHO groups. First strategy was according to International Diabetes Federation (IDF) parameter; adolescents with a minimum of two of these CMRFs were defined as MUO: (1) enhanced FBS (≥ 100 mg/dL), (2) increased TG (≥ 150 mg/dL), (3) reduced HDL-c (in boys: < 40 mg/dL/ in girls: < 50 mg/dL for those with 16 years and older, and < 40 mg/dL for those under 16 years), and (4) increased BP (≥ 130/85 mmHg) ^31^. In this strategy, adolescents with one or no CMRF were classified as MHO. Second strategy considered the presence of IR along with IDF parameters. Thus, adolescents with HOMA-IR ≥ 90th percentile (≥ 3.16) ^6,32^ and ≥ 2 of the mentioned CMRFs were stratified as MUO and those with HOMA-IR < 3.16 were determined as MHO, even if they had ≥ 2 CMRFs.

### Assessment of other variables

Physical activity (PA) levels of students were assessed via 9 items validated physical activity questionnaire for adolescents (PAQ-A) that scored from 1 (as lowest) to 5 (as highest) ^33^. The first 8 items of this questionnaire were related to common activities and the last parts was related to uncommon activity in the last week. After computing a total score, adolescents with a score of < 2, 2 ≤ score < 3, 3 ≤ score < 4, and score ≥ 4 were respectively categorized as sedentary (without any regular week activity), low active, moderately active, and very active. A standard checklist was applied to gather the students’ demographic data such as age, sex, history of illness, and taking drugs or nutritional supplements. Socioeconomic status (SES) was also evaluated via a validated questionnaire with the following parameters: parents' occupation and education level, family size, having personal room, having automobiles in the family, having laptops/computers, and traveling in the last year ^34^.

### Statistical analysis

The residual procedure was applied to calculate energy-adjusted DASH dietary components. After calculating the total DASH score for all adolescents, they were stratified based on energy-adjusted tertiles of DASH. General features of study subjects across tertiles of DASH were presented as mean ± SD/SE for continuous indicators and number and percentages for qualitative indicators. One-way analysis of variance (ANOVA) and chi-square tests were respectively applied to evaluate continuous and qualitative indicators across tertiles of DASH. Furthermore, total energy intake, age, and sex-adjusted dietary intakes of food groups and nutrients of subjects among tertiles of DASH were obtained via analysis of covariance (ANCOVA). To recognize relationship between tertiles of DASH and MUO, multivariable logistic regression was used. Odds ratios (ORs) and 95% confidence intervals (CIs) for DASH were calculated in crude and adjusted models. In the first model, adjustments were made for total energy intake, age, and sex. In the second one, additional adjustments were used for SES and physical activity levels. In the last model, BMI was added to the prior adjustments. In all models, the first tertile of DASH was assumed as the reference group. Tertiles of DASH were assumed as an ordinal variable to examine the trend of ORs. P values < 0.05 were considered as statistically significant. Version 20 of SPSS software was applied for all analyses.

### Ethical approval

The study procedure was performed according to declaration of Helsinki and STROBE checklist. All participants and their parents provided informed written consent. The study protocol was approved by the local Ethics Committee of Isfahan University of Medical Sciences.

### Consent to participate

Informed consent was obtained from all participants and their parents involved in the study.

## Results

In the present study, data of 203 adolescents (102 girls and 101 boys) with a mean (± SD) of 13.98 (± 1.61) years for age and 27.35 (± 3.24) kg/m^2^ for body mass index were assessed. Among participants, 42 girls and 37 boys (n = 79, 38.9%) suffered from MUO based on IDF definition, while 32 girls and 35 boys (n = 62, 33.0%) classified as MUO based on IDF/HOMA-IR criteria. General features and cardiometabolic factors of study subjects across energy-adjusted tertiles of DASH scores are depicted in Table 1. Subjects in the highest tertile of DASH diet had greater physical activity and HDL-c levels, as well as lower systolic and diastolic blood pressure, FBS, insulin, HOMA-IR, and TG levels, in comparison to the lowest tertile (all P values < 0.05).

**Table 1:** General characteristics and cardiometabolic factors of study participants across energy-adjusted tertiles of the DASH dietary score.

Tertiles of energy-adjusted DASH dietary score^a^				
	T1 (n = 67) (≤ 20)	T2 (n = 69) (21–27)	T3 (n = 67) (≥ 28)	P-value^b^
Sex, n (%)				0.77
Boys	36 (53.7)	34 (49.3)	32 (47.8)	
Girls	31 (46.3)	35 (50.7)	35 (52.2)	
Age (year)	14.19 $\pm$ 1.55	13.75 $\pm$ 1.43	13.99 $\pm$ 1.81	0.28
Weight (kg)	77.04 $\pm$ 11.18	72.52 $\pm$ 11.36	70.89 $\pm$ 11.55	0.06
Height (cm)	165.27 $\pm$ 8.78	163.17 $\pm$ 7.83	162.45 $\pm$ 6.97	0.10
BMI (kg/m^2^)	28.10 $\pm$ 2.63	27.12 $\pm$ 2.86	26.84 $\pm$ 3.98	0.05
Waist circumference (cm)	92.87 $\pm$ 6.81	89.76 $\pm$ 7.56	88.36 $\pm$ 8.75	0.01
Physical activity levels, n (%)				< 0.001
Sedentary and low active	66 (98.5)	64 (92.8)	36 (53.7)	
Moderately and very active	1 (1.5)	5 (7.2)	31 (46.3)	
Socioeconomic status^c^, n (%)				0.14
Low	27 (40.3)	18 (26.1)	14 (20.9)	
Medium	26 (38.8)	32 (46.4)	32 (47.8)	
High	14 (20.9)	19 (27.5)	21 (31.3)	
Systolic blood pressure (mmHg)	117.19 $\pm$ 10.11	112.30 $\pm$ 21.96	108.63 $\pm$ 19.83	0.02
Diastolic blood pressure (mmHg)	76.71 $\pm$ 5.87	72.21 $\pm$ 15.38	71.59 $\pm$ 10.08	0.01
Fasting blood glucose (mg/dL)	102.33 $\pm$ 9.06	98.48 $\pm$ 7.60	93.58 $\pm$ 6.37	< 0.001
Insulin (μUI/mL)	24.23 $\pm$ 14.62	21.23 $\pm$ 9.68	15.78 $\pm$ 11.93	< 0.001
HOMA-IR index	6.09 $\pm$ 3.57	5.21 $\pm$ 2.58	3.74 $\pm$ 3.22	< 0.001
Triglycerides (mg/dL)	147.16 $\pm$ 71.83	118.70 $\pm$ 67.25	100.09 $\pm$ 50.96	< 0.001
HDL-c (mg/dL)	42.48 $\pm$ 8.36	44.00 $\pm$ 6.62	48.01 $\pm$ 7.76	< 0.001

Values are Mean ± SD; unless indicated. Abbreviations: BMI: Body Mass Index; HOMA-IR: Homeostasis Model Assessment Insulin Resistance; HDL-c: high-density lipoprotein holesterol.

^a^Components of DASH diet were adjusted for total energy intake based on the residual method.

^b^P-value obtained from one-way ANOVA and χ2 test for quantitative and categorical variables, respectively.

^c^Socioeconomic status (SES) score was evaluated based on parental education level, parental job, number of family members, having computer/laptop, having personal room, having travel and having car in the family by using a validated questionnaire.

Dietary intakes of study subjects among tertiles of DASH diet are given in Table 2. Participants with the greatest accordance with DASH diet, in comparison to those with the lowest adherence, had remarkably greater intake of protein, potassium, total dietary fiber and cholesterol and lower intake of sodium. They also had a higher consumption of appropriate food groups for DASH diet (including vegetables, fruits, low fat dairy products, whole grains, and seeds, nuts & legumes) and a lower intake of inappropriate food groups for DASH diet (including red and processed meats and sweetened beverages). No considerable differences were seen in total energy intake, carbohydrate, fat, saturated fatty acids (SFA), monounsaturated fatty acids (MUFA) and polyunsaturated fatty acids (PUFA) consumption across tertiles of DASH diet.

**Table 2:** Multivariable-adjusted intakes of DASH diet components and selected nutrients of study participants across energy-adjusted tertiles of the DASH dietary score.

Tertiles of energy-adjusted DASH dietary score^a^				
	T1 (n = 67) (≤ 20)	T2 (n = 69) (21–27)	T3 (n = 67) (≥ 28)	P-value^b^
Energy, kcal	2975.53 $\pm$ 66.22	2817.93 $\pm$ 65.28	2857.54 $\pm$ 66.05	0.21
Nutrients				
Protein, % of energy	13.31 $\pm$ 0.22	14.34 $\pm$ 0.22	15.26 $\pm$ 0.22	< 0.001
Carbohydrate, % of energy	58.57 $\pm$ 0.63	58.46 $\pm$ 0.63	57.82 $\pm$ 0.63	0.66
Fat, % of energy	29.28 $\pm$ 0.63	28.70 $\pm$ 0.63	28.56 $\pm$ 0.63	0.69
Cholesterol, mg	256.53 $\pm$ 11.92	293.16 $\pm$ 11.70	296.17 $\pm$ 11.84	0.03
SFA, gr	27.46 $\pm$ 0.71	27.55 $\pm$ 0.70	27.04 $\pm$ 0.71	0.86
MUFA, gr	27.08 $\pm$ 0.84	27.31 $\pm$ 0.83	28.25 $\pm$ 0.83	0.58
PUFA, gr	29.92 $\pm$ 0.97	28.08 $\pm$ 0.96	27.46 $\pm$ 0.97	0.18
Potassium, mg	2642.77 $\pm$ 83.85	3402.00 $\pm$ 82.46	4087.94 $\pm$ 83.26	< 0.001
Sodium, mg	4452.79 $\pm$ 133.45	4018.59 $\pm$ 131.23	3493.97 $\pm$ 132.50	< 0.001
Total fiber, gr	14.94 $\pm$ 0.40	19.66 $\pm$ 0.39	23.72 $\pm$ 0.40	< 0.001
Food groups (g/day)				
Fruits	213.69 $\pm$ 16.67	343.53 $\pm$ 16.40	439.97 $\pm$ 16.56	< 0.001
Vegetables	161.10 $\pm$ 17.72	283.93 $\pm$ 17.43	399.88 $\pm$ 17.60	< 0.001
Whole grains	37.40 $\pm$ 10.82	72.67 $\pm$ 10.64	160.90 $\pm$ 10.74	< 0.001
Low fat dairy products	253.35 $\pm$ 21.14	362.64 $\pm$ 20.79	562.34 $\pm$ 20.99	< 0.001
Nuts, seeds and legumes	40.07 $\pm$ 3.52	60.96 $\pm$ 3.46	82.23 $\pm$ 3.49	< 0.001
Sweetened beverages	82.75 $\pm$ 5.52	44.79 $\pm$ 5.43	18.96 $\pm$ 5.48	< 0.001
Red and processed meats	33.83 $\pm$ 1.95	30.43 $\pm$ 1.92	19.07 $\pm$ 1.94	< 0.001

Values are Mean ± SE. Energy intake and macronutrients were adjusted for age and sex; all other values were adjusted for age, sex and energy intake. Abbreviations: SFA, Saturated fatty acids; MUFA, Monounsaturated fatty acids; PUFA, Polyunsaturated fatty acids.

^a^Components of DASH diet were adjusted for total energy intake based on the residual method.

^b^P-value obtained from ANCOVA test for adjustment of energy intake.

**Figure 1:**
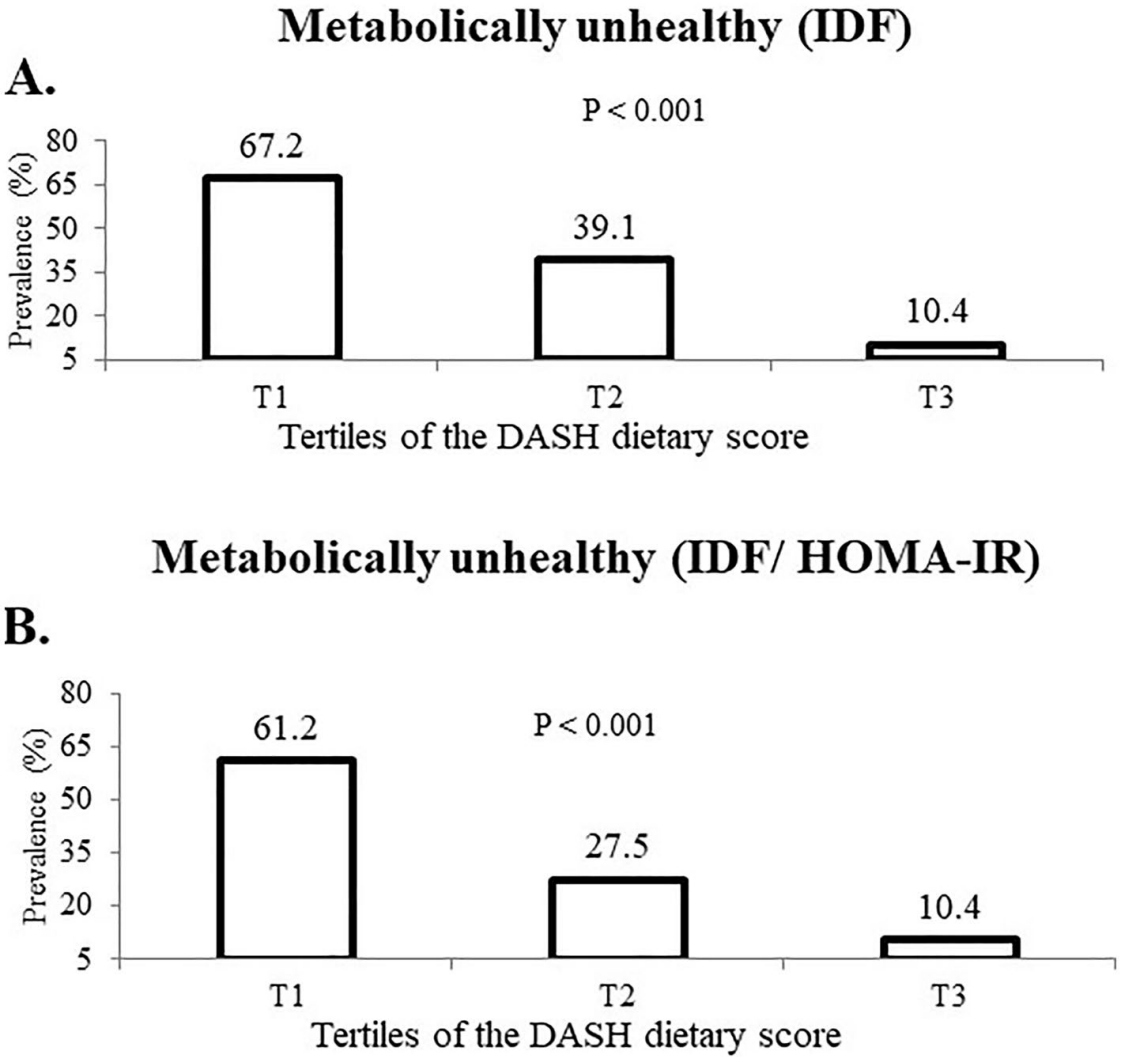
Prevalence of MUO based on IDF (**A**) and IDF/HOMA-IR criteria (**B**) in tertiles of DASH dietary pattern.

Prevalence of MUO phenotype based on both IDF and IDF/HOMA-IR criteria among tertiles of DASH diet is reported in Fig. 1. According to IDF definition, prevalence of MUO in the top tertile of DASH diet was lower than the bottom tertile (10.4 vs. 67.2%, P < 0.001). A similar finding was obtained when IDF/HOMA-IR criteria was applied (10.4 vs. 61.2%, P < 0.001).

Multivariate adjusted ORs and 95% CIs for MUO across energy-adjusted tertiles of DASH diet are provided in Table 3. Comparing top versus bottom tertile of DASH diet, there was a significantly inverse association between DASH diet and MUO odds, based on both IDF (OR = 0.06; 95% CI 0.02–0.15) and IDF/HOMA-IR definitions (OR = 0.07; 95% CI 0.03–0.19), in crude model. After adjustments for potential confounders, this relation did not substantially change. Such that, after considering all potential confounders, subjects in the third vs. first tertile of DASH diet had 92% and 91% lower odds of MUO based on IDF definition (OR = 0.08; 95% CI 0.03–0.22) and IDF/HOMA-IR criteria (OR = 0.09; 95% CI 0.03–0.29), respectively.

**Table 3:** Multivariate adjusted odds ratio (OR) and 95% confidence interval (CI) for MUO across energy-adjusted tertiles of the DASH dietary score.

	Tertiles of energy-adjusted DASH dietary score^a^			
	T1 (n = 67) (≤ 20)	T2 (n = 69) (21–27)	T3 (n = 67) (≥ 28)	P-trend
MUO phenotype based on IDF criteria				
Cases (n)	45	27	7	
Crude	1	0.31 (0.16–0.64)	0.06 (0.02–0.15)	< 0.001
Model 1	1	0.39 (0.19–0.80)	0.06 (0.02–0.16)	< 0.001
Model 2	1	0.42 (0.2–0.9)	0.08 (0.03–0.23)	< 0.001
Model 3	1	0.42 (0.20–0.90)	0.08 (0.03–0.22)	< 0.001
MUO phenotype based on IDF/HOMA-IR criteria				
Cases (n)	41	19	7	
Crude	1	0.24 (0.12–0.50)	0.07 (0.03–0.19)	< 0.001
Model 1	1	0.28 (0.13–0.60)	0.24 (0.10–0.56)	< 0.001
Model 2	1	0.31 (0.14–0.67)	0.10 (0.03–0.30)	< 0.001
Model 3	1	0.31 (0.14–0.68)	0.09 (0.03–0.29)	< 0.001

All values are odds ratios (OR) and 95% confidence intervals (CI). P-trend was obtained by the use of tertiles of DASH diet as an ordinal variable in the model. Model 1: Adjusted for age, sex, and total energy intake. Model 2: Additionally adjusted for physical activity and socioeconomic status (parental education, parental job, number of family members, having car in the family, having computer/laptop, having personal room and having trip). Model 3: Additionally adjusted for body mass index (BMI).

^a^Components of DASH diet were adjusted for total energy intake based on the residual method.

Multivariate adjusted ORs and 95% CIs for MUO across energy-adjusted tertiles of DASH diet among overweight versus obese adolescents are summarized in Table 4. According to IDF criteria, in crude and adjusted models, higher adherence to DASH diet was linked to significantly lower odds of MUO, both in overweight and obese subjects; but this odds reduction was stronger in overweight individuals. Such that, after considering all potential confounders, overweight adolescents in the third vs. first category of DASH diet had 99% (OR = 0.01; 95% CI 0.00–0.09) lower odds of MUO, while this odds reduction was 75% (OR = 0.25; 95% CI 0.07–0.95) in obese participants. The same pattern was observed when MUO was defined based on IDF/HOMA-IR parameters.

**Table 4:** Multivariate adjusted odds ratio (OR) and 95% confidence interval (CI) for MUO across energy-adjusted tertiles of the DASH dietary score, stratified by BMI categories.

Tertiles of energy-adjusted DASH dietary score^a^				
	T1 (≤ 20)	T2 (21–27)	T3 (≥ 28)	P-trend
MUO phenotype based on IDF criteria				
Overweight				
(Cases/participants)	17/24	10/35	1/45	
Crude	1	0.17 (0.05–0.52)	0.01 (0.00–0.08)	< 0.001
Model 1	1	0.19 (0.06–0.64)	0.01 (0.002–0.08)	< 0.001
Model 2	1	0.17 (0.05–0.65)	0.01 (0.00–0.09)	< 0.001
Obese				
(Cases/participants)	28/43	17/34	6/22	
Crude	1	0.54 (0.21–1.34)	0.20 (0.06–0.62)	0.01
Model 1	1	0.67 (0.25–1.74)	0.18 (0.05–0.63)	0.01
Model 2	1	0.75 (0.28–2.02)	0.25 (0.07–0.95)	0.06
MUO phenotype based on IDF/HOMA-IR criteria				
Overweight				
(Cases/participants)	14/24	5/35	1/45	
Crude	1	0.12 (0.03–0.41)	0.02 (0.00–0.14)	
Model 1	1	0.11 (0.03–0.43)	0.02 (0.00–0.15)	< 0.001
Model 2	1	0.11 (0.03–0.46)	0.03 (0.00–0.28)	< 0.001
Obese				
(Cases/participants)	27/43	14/34	6/22	< 0.001
Crude	1	0.41 (0.16–1.04)	0.22 (0.07–0.69)	0.01
Model 1	1	0.52 (0.20–1.35)	0.20 (0.06–0.70)	0.01
Model 2	1	0.56 (0.21–1.50)	0.25 (0.07–0.96)	0.06

All values are odds ratios (OR) and 95% confidence intervals (CI). P-trend was obtained by the use of tertiles of DASH diet as an ordinal variable in the model. Model 1: Adjusted for age, sex, and total energy intake. Model 2: Additionally adjusted for physical activity and socioeconomic status (parental education, parental job, number of family members, having car in the family, having computer/laptop, having personal room and having trip).

^a^Components of DASH diet were adjusted for total energy intake based on the residual method.

Stratified analysis by sex (Table 5) revealed that the relationship was stronger in girls (in crude model: OR = 0.03; 95% CI 0.00–0.14; and in fully adjusted model: OR = 0.02; 95% CI 0.00–0.13) than boys (in crude model: OR = 0.09; 95% CI 0.03–0.30; and in fully adjusted model: OR = 0.09; 95% CI 0.02–0.45), according to IDF criteria. Similar findings were obtained when MUO was determined based on IDF/HOMA-IR definition.

**Table 5:** Multivariate adjusted odds ratio (OR) and 95% confidence interval (CI) for MUO across energy-adjusted tertiles of the DASH dietary score, stratified by sex.

Tertiles of energy-adjusted DASH dietary score^a^				
	T1 (≤ 20)	T2 (21–27)	T3 (≥ 28)	P-trend
MUO phenotype based on IDF criteria				
Girl				
(Cases/participants)	25/36	15/34	2/32	
Crude	1	0.35 (0.13–0.93)	0.03 (0.00–0.14)	< 0.001
Model 1	1	0.47 (0.16–1.41)	0.01(0.00–0.11)	< 0.001
Model 2	1	0.46 (0.15–1.39)	0.02 (0.00–0.13)	< 0.001
Model 3	1	0.46 (0.15–1.39)	0.02 (0.00–0.13)	< 0.001
Boy				
(Cases/participants)	20/31	12/35	5/35	
Crude	1	0.29 (0.10–0.79)	0.09 (0.03–0.30)	< 0.001
Model 1	1	0.29 (0.10–0.83)	0.07 (0.02–0.29)	< 0.001
Model 2	1	0.39 (0.12–1.25)	0.12 (0.03–0.53)	0.01
Model 3	1	0.41 (0.12–1.34)	0.09 (0.02–0.45)	0.01
MUO phenotype based on IDF/HOMA-IR criteria				
Girl				
(Cases/participants)	22/36	8/34	2/32	
Crude	1	0.20 (0.07–0.55)	0.04 (0.01–0.21)	< 0.001
Model 1	1	0.24 (0.08–0.74)	0.03 (0.01–0.19)	< 0.001
Model 2	1	0.22 (0.07–0.69)	0.05 (0.01–0.31)	< 0.001
Model 3	1	0.22 (0.07–0.68)	0.05 (0.01–0.33)	< 0.001
Boy				
(Cases/participants)	19/31	11/35	5/35	
Crude	1	0.29 (0.10–0.80)	0.10 (0.03–0.35)	< 0.001
Model 1	1	0.31 (0.10–0.90)	0.09 (0.02–0.36)	< 0.001
Model 2	1	0.41 (0.12–1.32)	0.14 (0.03–0.65)	0.01
Model 3	1	0.42 (0.13–1.40)	0.11 (0.02–0.55)	0.01

All values are odds ratios (OR) and 95% confidence intervals (CI). P-trend was obtained by the use of tertiles of DASH diet as an ordinal variable in the model. Model 1: Adjusted for age, and total energy intake. Model 2: Additionally adjusted for physical activity and socioeconomic status (parental education, parental job, number of family members, having car in the family, having computer/laptop, having personal room and having trip). Model 3: Additionally adjusted for body mass index (BMI).

^a^Components of DASH diet were adjusted for total energy intake based on the residual method.

As indicated in Table 6, after considering all confounders, the greatest accordance with DASH diet versus the lowest adherence was respectively linked to a decline of 93%, 74%, 70% and 93% in odds of hyperglycemia (OR = 0.07; 95% CI 0.03–0.21), hypertriglyceridemia (OR = 0.26; 95% CI 0.09–0.73), low HDL-cholesterolemia (OR = 0.30; 95% CI 0.12–0.73) and insulin resistance (OR = 0.07; 95% CI 0.02–0.28).

**Table 6:** Multivariable adjusted odds ratio (OR) and 95% confidence interval (CI) for metabolic health components across energy-adjusted tertiles of the DASH dietary score.

Tertiles of energy-adjusted DASH dietary score^a^				
	T1 (n = 67) (≤ 20)	T2 (n = 69) (21–27)	T3 (n = 67) (≥ 28)	P-trend
High fasting blood glucose				
(Cases)	46	30	8	
Crude	1	0.35 (0.17–0.71)	0.06 (0.02–0.15)	< 0.001
Fully-adjusted model	1	0.34 (0.16–0.72)	0.07 (0.03–0.21)	< 0.001
High triglyceride				
(Cases)	28	16	8	
Crude	1	0.42 (0.20–0.88)	1.03 (0.61–1.73)	< 0.001
Fully-adjusted model^a^	1	0.47 (0.21–1.05)	0.26 (0.09–0.73)	0.01
Low HDL-c				
(Cases)	38	25	13	
Crude	1	0.43 (0.22–0.86)	0.18 (0.08–0.40)	< 0.001
Fully-adjusted model^a^	1	0.54 (0.26–1.13)	0.30 (0.12–0.73)	0.01
High blood pressure				
(Cases)	13	12	4	
Crude	1	0.87 (0.37–2.08)	0.26 (0.08–0.86)	0.03
Fully-adjusted model^a^	1	1.21 (0.47–3.10)	0.21 (0.04–1.02)	0.11
Insulin resistance				
(Cases)	62	59	25	
Crude	1	0.48 (0.15–1.47)	0.05 (0.02–0.13)	< 0.001
Fully-adjusted model^a^	1	0.60 (0.17–2.17)	0. 07 (0.02–0.28)	< 0.001

All values are odds ratios (OR) and 95% confidence intervals (CI).

HDL-c: high-density lipoprotein cholesterol. P-trend was obtained by the use of tertiles of DASH as an ordinal variable in the model. Fully-adjusted model: Adjusted for age, sex, total energy intake, physical activity, socioeconomic status, and body mass index (BMI).

^a^Components of DASH diet were adjusted for total energy intake based on the residual method.

## Discussion

In the present cross-sectional survey, a remarkable reverse relationship was shown between adherence to DASH diet and odds of MUO in Iranian adolescents according to two metabolic health definitions. This association remained notable, even after making adjustment for potential confounders. It should be highlighted that this association was stronger among girls and overweight adolescents in comparison to boys and subjects with obesity. Our findings on components of metabolic health status also indicated that the greatest compliance to DASH diet was linked to reduced odds of hyperglycemia, hypertriglyceridemia, low HDL-cholesterolemia and insulin resistance.

An MHO phenotype in adolescents might change to an MUO status and unfavorable clinical outcomes in adulthood ^35^. This conversion might lead to a greater risk of morbidity and mortality as well as reduced life expectancy in the future ^36^. Considering the inverse relationship observed between DASH diet and MUO phenotype in the present survey, it is clinically valuable to advise adolescents to increase consumption of health-related components of DASH diet including vegetables, fruits, low fat dairy products, whole grains, and nuts, seeds and legumes and reduce intake of unhealthy components such as sweetened beverages, salt and red and processed meats to enhance diet quality and reduce prevalence of metabolic disorders.

Few previous investigations have analyzed the relationship between DASH diet and MetS among children/ adolescents ^11,14^. The Tehran Lipid and Glucose study (TLGS), a prospective investigation with 3.6 years follow-up on 425 healthy adolescents with a mean age of 13.6 years ^11^, demonstrated that following DASH diet was reversely linked to incidence of MetS and some of its components, including abdominal obesity, hyperglycemia and hypertension ^11^. In another epidemiologic study on 628 participants aged 10–18 years, greater DASH scores were not linked to dyslipidemia hazard in adolescents, whereas DASH was inversely linked to risk of general and central obesity ^14^. In contrast, Park et al. have conducted a survey on individuals aged < 45 years and reported that there was no association between DASH diet and MHO phenotype ^19^. In another study on adults, Phillips et al. found that higher compliance with DASH diet was probably related to better metabolic health in non-obese subjects, but not in obese ones ^20^. In an observational study on overweight and obese Iranian children aged 6 to 13 years, greater compliance to DASH diet was associated with reduced odds of MUO based on insulin resistance; but no remarkable association was observed when MUO was defined based on cardio-metabolic risk factors ^8^. The observed inconsistencies among results of the above-mentioned investigations might be due to differences in age range of the study population, study design, criteria used to categorize MHO/MUO and potential confounders considered in the analyses.

A null relationship between DASH score and high blood pressure in the present study, similar to finding of Rahimi et al. study ^8^, could be attributed to two main reasons. First, notable effects of DASH dietary pattern on blood pressure might mostly occur in hypertensive subjects ^37^, not in people with somehow normal blood pressure values ^38,39^; only 14.2% (n = 29) of our study participants had high blood pressure. Second, the cut point of high blood pressure as a component of metabolic health status (BP ≥ 130/85 mmHg)^31^ was lower than hypertension definition (BP > 140/90 mmHg) ^40^.

So far, the precise mechanisms by which DASH diet might affect metabolic health status have not been identified. However, some possibilities have been proposed. A priori-defied DASH dietary pattern is rich in vegetables, fruits, whole grains, legumes, and nuts; therefore, abundant contents of fiber, antioxidants, potassium, and magnesium could have beneficial impacts on inflammation and metabolic profiles ^41^. Lower amounts of salt, refined sugar and saturated fat in DASH diet might also explain the favorable impacts of this diet on metabolic health ^17,42,43^. High fiber content could additionally elevate the generation of short-chain fatty acids (SCFAs) by gut microbiota ^44^. SCFAs could ameliorate metabolism of lipid and glucose in most of tissues ^44^. Moreover, antioxidants content could diminish oxidative stress by scavenging free radicals ^45^. The key role of oxidative stress in developing metabolic disorders is completely documented ^45^. Additionally, whole grains are related to a lower occurrence of insulin resistance because of a low glycemic index and slow absorption ^46^. Furthermore, the findings of previous surveys have shown inverse connections between calcium intake and FBS, lipid profiles, and blood pressure ^11,47,48^. The observed useful impacts of DASH diet might be due to a mixture of above-mentioned items ^49^.

Some strengths and limitations of this survey should be highlighted. First, to our knowledge, this is the first investigation that examined the linkage between DASH diet and metabolic health condition among Iranian adolescents with overweight and obesity. Second, two different criteria were applied to determine MUO/MHO phenotypes. Third, multiple confounders were controlled in the analyses. However, the design of the present survey was cross-sectional and did not permit us to conclude a causal relationship. So, conducting further prospective investigations is necessary. Although we evaluated BMI curves to categorize individuals into overweight and obese, we could not assess body composition and fat distribution, which could be substantial in recognizing metabolic health condition. Furthermore, misclassification of individuals was unavoidable due to applying FFQ for assessment of dietary intakes. Finally, despite controlling various covariates, remaining confounders (e.g., paternal obesity, birth weight, eating habits, and sleep disorders) might alter the findings.

In conclusion, findings of the current cross-sectional investigation demonstrated that greater compliance to DASH dietary pattern was linked to a remarkable lower odd of metabolic unhealthy condition among Iranian adolescents, especially in overweight subjects and girls. More prospective studies on a larger scale from different countries are necessary to affirm these findings.

## Data Availability

Supporting data for the findings of this investigation are available from the corresponding author (PS) upon reasonable request.

## Abbreviations

ANOVA: Analysis of variance
ANCOVA: Analysis of covariance
FFQ: Food frequency questionnaire
OR: Odds ratios
95% CI: 95% Confidence interval
BMI: Body mass index
MHO: Metabolically healthy obesity
MUO: Metabolically unhealthy obesity
IDF: International Diabetes Federation
HOMA-IR: Homeostasis model assessment for insulin resistance
PAQ-A: Physical activity questionnaire for adolescents
SES: Socioeconomic status
DASH: Dietary approaches to stop hypertension
WHO: World Health Organization
USDA: United States Department of Agriculture
MUFA: Mono unsaturated fatty acids
PUFA: Poly unsaturated fatty acids
SFA: Saturated fatty acids
SCFA: Short-chain fatty acids
WC: Waist circumference
SBP: Systolic blood pressure
DBP: Diastolic blood pressure
TG: Triglycerides
HDL-c: High-density lipoprotein cholesterol
FBS: Fasting blood sugar
SPSS: Statistical package for social sciences

## References

[1] Fryar, C. D., Carroll, M. D. & Ogden, C. L. Prevalence of overweight, obesity, and severe obesity among children and adolescents aged 2–19 years: United States, 1963–1965 through 2015–2016 (2018).

[2] Asgari E, Askari M, Bellissimo N, Azadbakht L (2022). Association between ultraprocessed food intake and overweight, obesity, and malnutrition among children in Tehran, Iran. Int. J. Clin. Pract..

[3] Lobstein T, Jackson-Leach R (2016). Planning for the worst: Estimates of obesity and comorbidities in school-age children in 2025. Pediatr. Obes..

[4] Horesh A, Tsur AM, Bardugo A, Twig G (2021). Adolescent and childhood obesity and excess morbidity and mortality in young adulthood—A systematic review. Curr. Obes. Rep..

[5] Drozdz D, Alvarez-Pitti J, Wójcik M, Borghi C, Gabbianelli R, Mazur A (2021). Obesity and cardiometabolic risk factors: From childhood to adulthood. Nutrients..

[6] Prince RL, Kuk JL, Ambler KA, Dhaliwal J, Ball GD (2014). Predictors of metabolically healthy obesity in children. Diabetes Care..

[7] Vukovic R, Dos Santos TJ, Ybarra M, Atar M (2019). Children with metabolically healthy obesity: A review. Front. Endocrinol..

[8] Rahimi H, Yuzbashian E, Zareie R, Asghari G, Djazayery A, Movahedi A (2020). Dietary approaches to stop hypertension (DASH) score and obesity phenotypes in children and adolescents. Nutr. J..

[9] Roberge J-B, Van Hulst A, Barnett TA, Drapeau V, Benedetti A, Tremblay A (2019). Lifestyle habits, dietary factors, and the metabolically unhealthy obese phenotype in youth. J. Pediatr..

[10] Sacks FM, Svetkey LP, Vollmer WM, Appel LJ, Bray GA, Harsha D (2001). Effects on blood pressure of reduced dietary sodium and the Dietary Approaches to Stop Hypertension (DASH) diet. N. Engl. J. Med..

[11] Asghari G, Yuzbashian E, Mirmiran P, Hooshmand F, Najafi R, Azizi F (2016). Dietary approaches to stop hypertension (DASH) dietary pattern is associated with reduced incidence of metabolic syndrome in children and adolescents. J. Pediatr..

[12] Bricarello LP, de Almeida Alves M, Retondario A, de Moura Souza A, de Vasconcelos FDAG (2021). DASH diet (Dietary Approaches to Stop Hypertension) and overweight/obesity in adolescents: The ERICA study. Clin. Nutr. ESPEN..

[13] Cohen JF, Lehnerd ME, Houser RF, Rimm EB (2017). Dietary approaches to stop hypertension diet, weight status, and blood pressure among children and adolescents: National Health and Nutrition Examination Surveys 2003–2012. J. Acad. Nutr. Diet..

[14] Farhadnejad H, Asghari G, Mirmiran P, Azizi F (2018). Dietary approach to stop hypertension diet and cardiovascular risk factors among 10-to 18-year-old individuals. Pediatr. Obes..

[15] Golpour-Hamedani S, Mohammadifard N, Khosravi A, Feizi A, Safavi SM (2017). Dietary approaches to stop hypertension diet and obesity: A cross-sectional study of Iranian children and adolescents. ARYA Atheroscler..

[16] Hajna S, Liu J, LeBlanc PJ, Faught BE, Merchant AT, Cairney J (2012). Association between body composition and conformity to the recommendations of Canada's Food Guide and the Dietary Approaches to Stop Hypertension (DASH) diet in peri-adolescence. Public Health Nutr..

[17] Saneei P, Hashemipour M, Kelishadi R, Rajaei S, Esmaillzadeh A (2013). Effects of recommendations to follow the Dietary Approaches to Stop Hypertension (DASH) diet v. usual dietary advice on childhood metabolic syndrome: A randomised cross-over clinical trial. Br. J. Nutr..

[18] Bricarello LP, Poltronieri F, Fernandes R, Retondario A, de Moraes Trindade EBS, de Vasconcelos FDAG (2018). Effects of the Dietary Approach to Stop Hypertension (DASH) diet on blood pressure, overweight and obesity in adolescents: A systematic review. Clin. Nutr. ESPEN..

[19] Park Y-MM, Steck SE, Fung TT, Zhang J, Hazlett LJ, Han K (2017). Mediterranean diet, Dietary Approaches to Stop Hypertension (DASH) style diet, and metabolic health in US adults. Clin. Nutr..

[20] Phillips CM, Dillon C, Harrington JM, McCarthy VJ, Kearney PM, Fitzgerald AP (2013). Defining metabolically healthy obesity: Role of dietary and lifestyle factors. PLoS ONE..

[21] Qorbani M, Khashayar P, Rastad H, Ejtahed H-S, Shahrestanaki E, Seif E (2020). Association of dietary behaviors, biochemical, and lifestyle factors with metabolic phenotypes of obesity in children and adolescents. Diabetol. Metab. Syndr..

[22] Yaghoubpour K, Tasdighi E, Abdi H, Barzin M, Mahdavi M, Valizadeh M (2021). Association of obesity phenotypes in adolescents and incidence of early adulthood type 2 diabetes mellitus: Tehran lipid and glucose study. Pediatr. Diabetes..

[23] Onis MD, Onyango AW, Borghi E, Siyam A, Nishida C, Siekmann J (2007). Development of a WHO growth reference for school-aged children and adolescents. Bull. World Health Organ..

[24] Kelishadi R, Majdzadeh R, Motlagh M-E, Heshmat R, Aminaee T, Ardalan G (2012). Development and evaluation of a questionnaire for assessment of determinants of weight disorders among children and adolescents: The Caspian-IV study. Int. J. Prev. Med..

[25] Daneshzad E, Ghorabi S, Hasani H, Omidian M, Pritzl TJ, Yavari P (2019). Food insecurity is positively related to Dietary Inflammatory Index in Iranian high school girls. Int. J. Vitamin Nutr. Res..

[26] Mohseni H, Mohammadi FM, Karampour Z, Amini S, Abiri B, Sayyah M (2021). The relationship between history of dietary nutrients intakes and incidence of aggressive behavior in adolescent girls: A case–control study. Clin. Nutr ESPEN..

[27] Ghaffarpour M, Houshiar-Rad A, Kianfar H (1999). The manual for household measures, cooking yields factors and edible portion of foods. Tehran Nashre Olume Keshavarzy..

[28] Challa, H. J., Ameer, M. A., & Uppaluri, K. R. DASH Diet To Stop Hypertension. StatPearls. Treasure Island (FL) ineligible companies. Disclosure: Muhammad Atif Ameer declares no relevant financial relationships with ineligible companies. Disclosure: Kalyan Uppaluri declares no relevant financial relationships with ineligible companies.: StatPearls Publishing Copyright © 2024, (StatPearls Publishing LLC., 2024).

[29] Benisi-Kohansal S, Shayanfar M, Mohammad-Shirazi M, Tabibi H, Sharifi G, Saneei P (2016). Adherence to the dietary approaches to stop hypertension-style diet in relation to glioma: A case–control study. Br. J. Nutr..

[30] Swaroop JJ, Rajarajeswari D, Naidu J (2012). Association of TNF-α with insulin resistance in type 2 diabetes mellitus. Indian J. Med. Res..

[31] Rvoets L, Massa G (2016). Classification and clinical characterization of metabolically “healthy” obese children and adolescents. J. Pediatr. Endocrinol. Metab..

[32] Keskin M, Kurtoglu S, Kendirci M, Atabek ME, Yazici C (2005). Homeostasis model assessment is more reliable than the fasting glucose/insulin ratio and quantitative insulin sensitivity check index for assessing insulin resistance among obese children and adolescents. Pediatrics..

[33] Kowalski KC, Crocker PR, Donen RM (2004). The physical activity questionnaire for older children (PAQ-C) and adolescents (PAQ-A) manual. Coll. Kinesiol. Univ. Saskatchewan..

[34] Garmaroudi GR, Moradi A (2010). Socio-economic status in Iran: A study of measurement index. Payesh.

[35] Schröder H, Ramos R, Baena-Díez JM, Mendez MA, Canal DJ, Fíto M (2014). Determinants of the transition from a cardiometabolic normal to abnormal overweight/obese phenotype in a Spanish population. Eur. J. Nutr..

[36] Goday A, Calvo E, Vázquez LA, Caveda E, Margallo T, Catalina-Romero C (2016). Prevalence and clinical characteristics of metabolically healthy obese individuals and other obese/non-obese metabolic phenotypes in a working population: Results from the Icaria study. BMC Public Health..

[37] Nowson CA, Wattanapenpaiboon N, Pachett A (2009). Low-sodium Dietary Approaches to Stop Hypertension–type diet including lean red meat lowers blood pressure in postmenopausal women. Nutr. Res..

[38] Li Y, Yang Y, Ju L, Piao W, Wei X, Zhao L (2023). Association of the protective dietary pattern for blood pressure with elevated blood pressure and hypertension among Chinese children and adolescents aged 6–17 years old: Data from China nutrition and health surveillance (2015–2017). Nutrients..

[39] Hodson L, Harnden K, Roberts R, Dennis A, Frayn K (2010). Does the DASH diet lower blood pressure by altering peripheral vascular function?. J. Hum. Hypertens..

[40] Judd E, Calhoun DA (2014). Apparent and true resistant hypertension: Definition, prevalence and outcomes. J. Hum. Hypertens..

[41] Saneei P, Hashemipour M, Kelishadi R, Esmaillzadeh A (2014). The Dietary Approaches to Stop Hypertension (DASH) diet affects inflammation in childhood metabolic syndrome: A randomized cross-over clinical trial. Ann. Nutr. Metab..

[42] Esmaillzadeh A, Azadbakht L (2008). Consumption of hydrogenated vs. non-hydrogenated vegetable oils and risk of insulin resistance and the metabolic syndrome among Iranian adult women. Diabetes Care..

[43] Golzarand M, Moslehi N, Mirmiran P, Azizi F (2023). Adherence to the DASH, MeDi, and MIND diet scores and the incidence of metabolically unhealthy phenotypes. Obes. Res. Clin. Pract..

[44] Partula V, Deschasaux M, Druesne-Pecollo N, Latino-Martel P, Desmetz E, Chazelas E (2020). Associations between consumption of dietary fibers and the risk of cardiovascular diseases, cancers, type 2 diabetes, and mortality in the prospective NutriNet-Santé cohort. Am. J. Clin. Nutr..

[45] Harasym J, Oledzki R (2014). Effect of fruit and vegetable antioxidants on total antioxidant capacity of blood plasma. Nutrition..

[46] De Munter JSL, Hu FB, Spiegelman D, Franz M, Van Dam RM (2007). Whole grain, bran, and germ intake and risk of type 2 diabetes: A prospective cohort study and systematic review. PLoS Med..

[47] Sahebkar A, Heidari Z, Kiani Z, Atefi M, Zareie A, Shojaei M (2023). The efficacy of dietary approaches to stop hypertension (DASH) diet on lipid profile: A systematic review and meta-analysis of clinical controlled trials. Curr. Med. Chem..

[48] Thomas AP, Dunn TN, Drayton JB, Oort PJ, Adams SH (2013). A dairy-based high calcium diet improves glucose homeostasis and reduces steatosis in the context of preexisting obesity. Obesity..

[49] Al-Solaiman Y, Jesri A, Mountford WK, Lackland DT, Zhao Y, Egan BM (2010). DASH lowers blood pressure in obese hypertensives beyond potassium, magnesium and fibre. J. Hum. Hypertens..

